
JOURNAL INFORMATION
==============================
NLM Title Abbreviation: Innov Aging
Journal ID: Innov Aging
NLM Journal ID: 101703706
Journal ID: innovateage

Innovation in Aging

EISSN: 2399-5300
Publisher: Oxford University Press

ARTICLE INFORMATION
==============================
PMCID: PMC6840060
DOI: 10.1093/geroni/igz038.1053
Article ID: igz038.1053
Article version: 1
Subjects: Session 1355 (Poster), Family Caregiving I

THE EFFECT OF CAREGIVING ON THE DEPRESSIVE SYMPTOMS OF OLDER SPOUSAL CAREGIVERS IN CHINA

Zhao Xinyi 1
Liu Huiying 2
Wang Zhaoguang 3
Zhang Quan 4
Lou Vivian W 5
1 School of Health Humanities , Peking University, Beijing, China
2 University of Hong Kong , Hong Kong, Hong Kong
3 National School of Development, Peking University , Beijing, Beijing, China
4 Peking University Health Science Center , Beijing, China, China
5 The University of Hong Kong , Hong Kong, P.R.C., Hong Kong
Publication date: 2019 Nov
Electronic publication date: 2019 Nov 8
Volume: 3
Issue: Suppl 1
Issue title: Program Abstracts from the GSA 2019 Annual Scientific Meeting “Strength in Age—Harnessing the Power of Networks”
First page: S285
Last page: S285
Copyright: © The Author(s) 2019. Published by Oxford University Press on behalf of The Gerontological Society of America.
Copyright year: 2019
License: This is an Open Access article distributed under the terms of the Creative Commons Attribution License (http://creativecommons.org/licenses/by/4.0/), which permits unrestricted reuse, distribution, and reproduction in any medium, provided the original work is properly cited.
License URL: https://creativecommons.org/licenses/by/4.0/

Page count: 1

==============================
### THE EFFECT OF CAREGIVING ON THE DEPRESSIVE SYMPTOMS OF OLDER SPOUSAL CAREGIVERS IN CHINA

Xinyi Zhao,[1] Huiying Liu,[2] Zhaoguang Wang,[3] Quan Zhang,[4] and Vivian W. Lou[5], *1. School of Health Humanities, Peking University, Beijing, China, 2. University of Hong Kong, Hong Kong, Hong Kong, 3. National School of Development, Peking University, Beijing, Beijing, China, 4. Peking University Health Science Center, Beijing, China, China, 5. The University of Hong Kong, Hong Kong, P.R.C., Hong Kong*

Objectives: This study investigated the influence of caregiving on the depressive symptoms of older spousal caregivers in China. Methods: Data came from the 2015 China Health and Retirement Longitudinal Study (CHARLS). The sample of this study included older adults aged 60 and above. The respondents were coded as spousal caregivers if they provided activities of daily life (ADL)/instrumental activities of daily life (IADL) assistance to their spouses and did not receive any ADL/IADL help. Ordinary Least Square (OLS) regression analyses were applied. Results: Results from the regression model indicated that spousal caregiving was significantly associated with depressive symptoms (p<0.01). The spousal caregivers showed more depressive symptoms than the non-caregivers. Among the spousal caregivers, the females and those who reported poorer health had more depressive symptoms than their counterparts (p<0.01). Conclusion: The results highlight the negative influence of caregiving on psychological health of spousal caregivers. Caregiver support should be targeted on the older spousal caregivers, especially the wife caregivers.
